# Characterisation of the attenuation properties of 3D-printed tungsten for use in gamma camera collimation

**DOI:** 10.1186/s40658-018-0238-3

**Published:** 2019-01-07

**Authors:** Jonathan I. Gear, Jan Taprogge, Owen White, Glenn D. Flux

**Affiliations:** Joint Department of Physics, Royal Marsden NHSFT and Institute of Cancer Research, Downs Road, Sutton, SM2 5PT UK

**Keywords:** Collimator, Monte Carlo, 3D printing, Selective laser melting, Tungsten

## Abstract

**Background:**

The aim of this work was to characterise the attenuation properties of 3D-printed tungsten and to assess the feasibility for its use in gamma camera collimator manufacture.

**Method:**

3D-printed tungsten disks were produced using selective laser melting (SLM). Measurements of attenuation were made through increasing numbers of disks for a Tc-99m (140 keV) and I-131 (364 keV) source. The technique was validated by repeating the measurements with lead samples. Resolution measurements were also made with a SLM tungsten collimator and compared to Monte Carlo simulations of the experimental setup. Different collimator parameters were simulated and compared against the physical measurements to investigate the effect on image quality.

**Results:**

The measured disk thicknesses were on average 20% above the specified disk thicknesses. The measured attenuation for the tungsten samples were lower than the theoretical value determined from the National Institute of Standards and Technology (NIST) cross-sectional database (Berger and Hubbell, XCOM: photon cross-sections on a personal computer, 1987). The laser scan strategy had a significant influence on material attenuation (up to 40% difference). Results of these attenuation measurements indicate that the density of the SLM material is lower than the raw tungsten density. However, an improved performance compared to a lead collimator was observed. The SLM tungsten collimator was adequately simulated as 80% density and 110% septal thickness. Scatter and septal penetration were 17% less than a similar lead collimator and 33% greater than tungsten at 100% density.

**Conclusions:**

SLM manufacture of tungsten collimators is feasible. Attenuation properties of SLM tungsten are superior to the lead alternative and the opportunity for bespoke collimator design is appealing.

## Background

### Selective laser melting

Rapid prototyping (also known as solid freeform fabrication, additive manufacturing and 3D printing) has been available for almost 30 years [1], primarily for manufacturing scale models and product prototypes. Recent advances have seen a rapid growth in its application with desktop and home systems now available. Printers are generally based on three main techniques; thermos extrusion, powder deposition and stereolithography. Despite being more commonly used to generate plastic parts, additive manufacturing techniques can also be employed using other materials such as paper, wood, ceramic, glass and a variety of metals.

Selective laser melting (SLM) using powder deposition techniques are the most common methods employed for generating metal objects. A high-power laser is used to fuse small particles of metal powders into a mass that has a desired three-dimensional shape. The laser selectively fuses powdered material by scanning cross-sections of the part on the surface of a powder bed. After each cross-section is scanned, the powder bed is lowered by one layer thickness, a new layer of material is applied on top via an additional supply bed and roller and the process is repeated until the part is completed.

The density of the finished part depends on gaps between the sintered material generated during manufacture. These can be reduced by adjusting peak laser power, scan speed, hatch spacing, particle size and chamber pressure. Reported densities for SLM vary depending on the geometry and complexity of the finished piece and can range from 80 to 96% of the theoretical density [2–4].

### 3D tungsten collimator printing

Due to its high density (19.25 g/cm^3^), tungsten is an appealing material for use in radiation shielding. The half value layer of tungsten is approximately 25% less than that of lead at the energies used in nuclear medicine, hence tungsten is often used for manufacture of syringe shields and Mo-99 generator casing. Tungsten may also be used for the manufacture of gamma camera collimators, whereby the higher stopping power of tungsten could offer more flexibility in collimator optimization either through enhanced sensitivity or spatial resolution. However, due to the high melting point of tungsten compared to lead (3422 °C to 328 °C) and the increased raw material cost (× 200%), conventional manufacturing techniques are prohibitively costly.

SLM could help to overcome the difficulty in tungsten collimator manufacturing and enable a more cost-effective solution for bespoke manufacture. A number of commercial enterprises are currently offering SLM of tungsten (M&I Materials UK, Smit Röntgen, NL, Layerwise, Belgium) and a few conceptual collimator designs have been proposed. Applications for tungsten collimators have been proposed for high energy I-131 imaging [5, 6] and in SPECT/MR systems to reduce Eddie currents [7–9]. Due to the novelty of this technology, an extensive investigation of the expected attenuation and production accuracy of SLM tungsten during collimator production has not been undertaken. The aim of this study was to investigate the attenuation and manufacturing accuracy of SLM tungsten (Wolfmet of M&I Materials Ltd. Hibernia Way, Trafford Park, Manchester, UK) and inform on the optimal print strategy for collimator production. Samples of SLM tungsten were printed at sub-millimetre thicknesses (typical of gamma camera collimators) for different printing strategies in a printing orientation equal to that of collimator septa. Direct measurements of the attenuation of the disks are made and the test validated against those of lead. Results of the attenuation experiments are used to estimate the performance and manufacturing characteristics of an SLM collimator which is then compared to results generated using Monte Carlo simulations.

## Method

### Theoretical determination of the attenuation coefficient

Theoretical values for the attenuation coefficient of both lead and tungsten were calculated using the National Institute of Standards and Technology (NIST) cross-sectional database (XCOM) [10] and the reported density of the raw metal. The NIST database was constructed through the combination of incoherent and coherent scattering cross sections. Incoherent (Compton) scattering cross sections were obtained from a combination of the Klein-Nishina formula and non-relativistic Hartree-Fock incoherent scattering functions. The coherent (Rayleigh) scattering cross sections were calculated from a combination of the Thompson formula and relativistic Hartree-Fock atomic form factors. The photoelectric cross sections were obtained by a phase-shift calculation for a central potential and a Hartree-Slater atomic model.

### SLM manufacture

Five sets (labelled A–E) of six tungsten disks were manufactured with central thicknesses of 0.3, 0.5 and 1.2 mm on an EOS M280 powder bed laser fusion system (EOS GmbH, Munich, Germany). Printing was operated at maximum available laser power, 370 W, with an 80 μm laser spot diameter, operated in continuous wave mode. The thickness of the deposited powder layer was set to 30 μm in this study and the printing chamber flooded with argon gas to reduce oxygen to < 100 ppm.

**Fig. 1:**
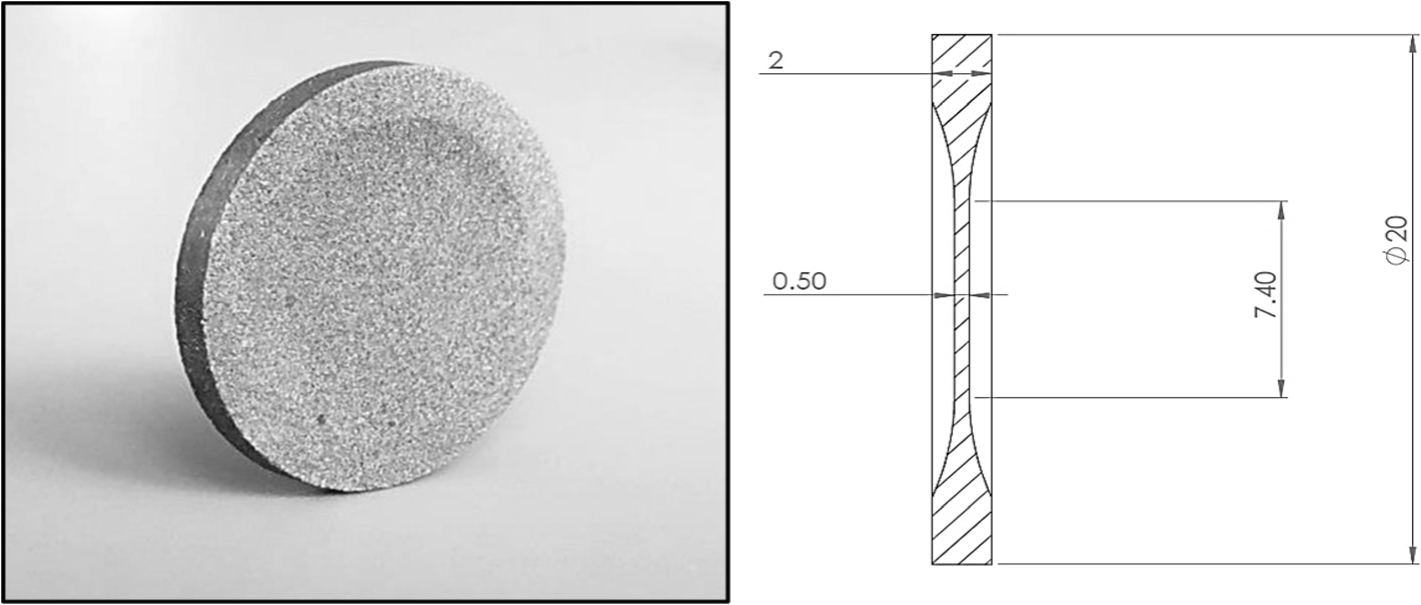
Photograph of SLM disk and schematic diagram of disk dimensions

For handling ease and to allow removal from the printing plate, a 2.0 mm tapered annulus was included around the periphery of each disk. A photograph of a typical disk and schematic cross-section in the printing plane are given in Fig. 1. Disks were printed in an orientation such that the central section was orientated in the same printing plane as collimator septa.

Production accuracy was assessed against micrometre measurements of the central disk section using a digital display micrometre (Mitutoyo, 293-766-50) with a product measurement accuracy of ± 2 μm. For each disk set, different scan strategies were investigated, and summarised in Table 1.

**Table 1:**
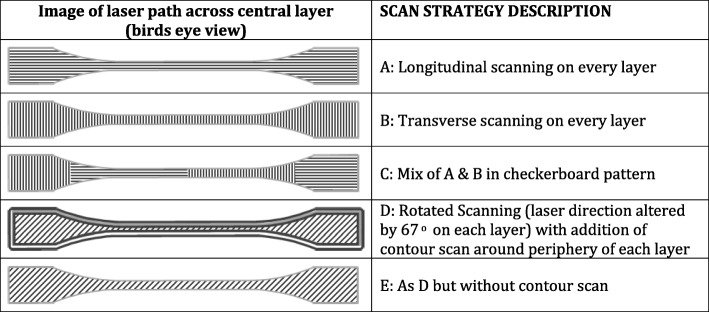
Scan strategies employed in the productionof the SLM tungsten disks

### Attenuation measurements

**Fig. 2:**
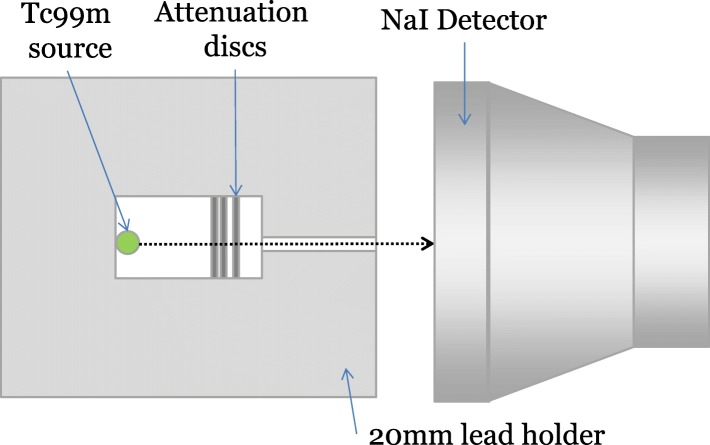
Schematic diagram of attenuation measurements

Measurements of attenuation were made by placing an increasing number of disks from each sample set within a 20-mm-thick lead holder, housing a radioactive point source. A 1-mm-diameter hole collimated the photons from the source at a 200-mm-diameter NaI(Tl) detector positioned 50 mm from the aperture and connected to a signal amplifier and multi-channel analyser. A schematic of the set-up is shown in Fig. 2.

The total tungsten thickness (based on the micrometre measurements of the previous section) between the source and collimator hole varied from 0 to 3.4 mm. Acquisitions were also performed using 0.5 mm thick lead disks, with Tc-99m (140 keV) and I-131 (364 keV) point sources to assess the attenuation properties of both materials. Energy spectra of the collimated beam were generated with 300-s acquisitions. Spectra were corrected for background and scatter by subtracting spectra data acquired in a similar geometry with the absence of the 1 mm collimation hole. For each energy spectra, the net counts within a 20% energy window centred at the photopeak were measured and corrected for deadtime and radioactive decay. Net decay-corrected count rate verses disk thickness was plotted on a log-linear scale and the gradient of the fit to the data was used to determine the material attenuation coefficient, μ, such that:

$$ {C}_t={C}_0.{e}^{-\mu .t} $$

where *C*_*0*_ is the non-attenuated count rate and *C*_*t*_ is the attenuated count rate of material thickness *t*.

### Collimator measurements

Performance characteristics of an SLM tungsten collimator designed for small animal Tc99m planar imaging were assessed using scintigraphy imaging on a Siemens Symbia Intevo gamma camera (Erlangen Germany). The 10-mm-thick collimator measured 55 × 55 mm and comprised 1380 1.3 mm hexagonal parallel holes with nominal septal thickness of 0.2 mm. The collimator was manufactured using the same printing method described for the disks. A rotated scanning strategy was employed with the addition of a contour scan around periphery of each layer (equivalent to scan strategy D).

**Fig. 3:**
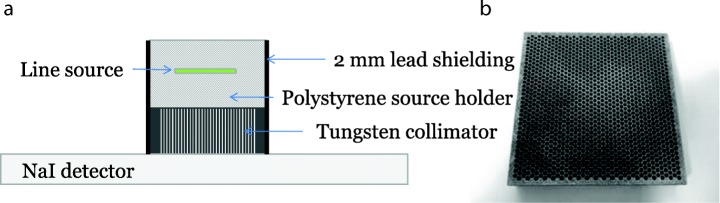
Schematic diagram of collimator measurement

The tungsten collimator was placed on the surface of the camera crystal and a 30 mm 10 MBq Tc-99m line source (1 mm diameter) placed 15, 30, 45, 60 and 75 mm from the collimator. A lead mask was then placed around both source and collimator to reduce scatter events outside the collimator field of view. For each source location, static acquisitions were acquired with a × 3 image magnification and 1024 matrix size, giving a final image pixel size of 0.19 mm. Acquisitions were acquired for 300 s. Summed profiles across the length of the line source were generated using ImageJ. A photograph of the collimator and schematic of the setup is shown in Fig. 3.

### Monte Carlo

Monte Carlo simulations of the experimental measurements were performed using the SIMIND Monte Carlo software [11] which allows the user to specify input criteria for the geometry and material for the source, phantom, collimator and detector. Simulations of the collimator measurements were performed using input criteria that matched the Siemens Symbia gamma camera. Input data for crystal thickness, backscatter layer, intrinsic energy and intrinsic spatial resolution were based on validated input criteria from an earlier study [12] and summarised in Table 2.

**Table 2:** SIMIND input parameters for simulation of a Siemens Symbia Gamma Camera Detector

SIMIND input	Value
Crystal thickness (NaI(Tl))	9.5 (mm)
Backscatter Thickness (PMT)	11.0 (cm)
Cover thickness (Al)	0.1 (mm)
Gap between collimator and detector	1.0 (mm)
Energy resolution (@ 140 keV)	9.0 (%)
Intrinsic spatial resolution (@ 140 keV)	3.6 (mm)
Max. scatter order	10
Cut off energy	1.0 keV

Changes to the simulation inputs for the standard gamma camera collimator were made to match the specifications of the Tungsten collimator and a 1-mm-diameter line source positioned across the full length of the detector field of view. Two hundred million isotopically distributed 140 keV photons were simulated. Events from primary, scattered and septal penetrating photons incident on the detector were recorded. Summed profiles across the length of the generated line source images were generated using ImageJ and compared to experimental measurements. The collimator material density value contained within the SIMIND cross-section file for tungsten was altered for decreasing levels of density (100–70%) and the analysis of the simulation data repeated with different septal thicknesses (± 20%); these values were chosen based on the observed results obtained for the SLM disks.

## Results

### Theoretical determination of the attenuation coefficient

Attenuation as a fraction of coherent, incoherent and photoelectric absorption for lead and tungsten are summarised in Tables 3 and 4. For the experimental measurements, it is recognised that coherent scatter events cannot be distinguished from true penetration events and therefore a small proportion of low-angled scatter events may be included within the measured attenuation coefficient. Expected measured attenuation is therefore likely to be slightly lower than the theoretical measurement, but should not be less than the total contribution from photoelectric and incoherent scatter events (μ > 34.29 cm^−1^ for tungsten and μ > 25.77 cm^−1^ for lead).

**Table 3:** Theoretical linear attenuation coefficients of tungsten at photopeak energies of Tc99m and I131 organised by interaction

Tungsten	140 keV		364 keV	
Interaction	% contribution	μ (cm^−1^)	% contribution	μ (cm^−1^)
Incoherent	5.2	1.90	34.1	1.48
Coherent	5.3	1.93	7.8	0.34
Photoelectric	89.5	32.39	58.1	2.52
Total	–	36.23	–	4.34

**Table 4:** Theoretical linear attenuation coefficients of lead at photopeak energies of Tc99m and I131 organised by interaction

Lead	140 keV		364 keV	
Interaction	% contribution	μ (cm^−1^)	% contribution	μ (cm^− 1^)
Incoherent	4.0	1.09	27.4	0.86
Coherent	5.0	1.34	7.6	0.24
Photoelectric	91.0	24.68	65.0	2.03
Total	–	27.11	–	3.12

### Lead samples

**Fig. 4:**
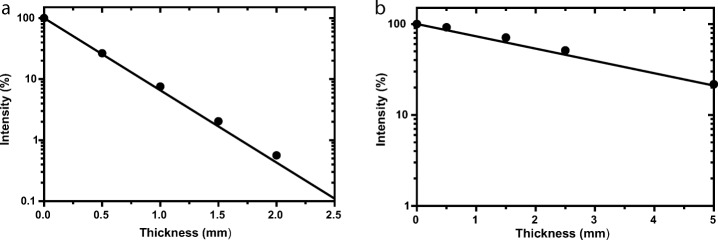
Measured beam intensity of Tc99m source (**a**) and I-131 source (**b**) through different thicknesses of lead. The theoretical ideal is represented as the solid black line of each plot

Plots of the normalised count rate (intensity) as a function of total lead disk thickness measured at 140 keV and 364 keV are shown on log-linear plots in Fig. 4. The linear attenuation coefficient is measured as the gradient of the fits to the data and are summarised in Table 5 with comparison to the theoretical ideal (solid line). For each energy, the measurement uncertainty range fell within the expected theoretical range and therefore validated the measurement technique.

**Table 5:** Measured attanution cooficients of lead and comparison to theoretical

	Measured μ (cm^−1^)	% deviation from theoretical (including Coherent scatter)	% deviation from theoretical (excluding Coherent scatter)
Lead disks @ 140 keV	26.0 ± 0.2	− 4.1%	0.9%
Lead disks @ 364 keV Tungsten	2.8 ± 0.2	− 9.6%	− 2.1%

The linear attenuation coefficients of lead measured at 140 keV and 364 keV are summarised in Table 5.

### SLM manufacture

**Fig. 5:**
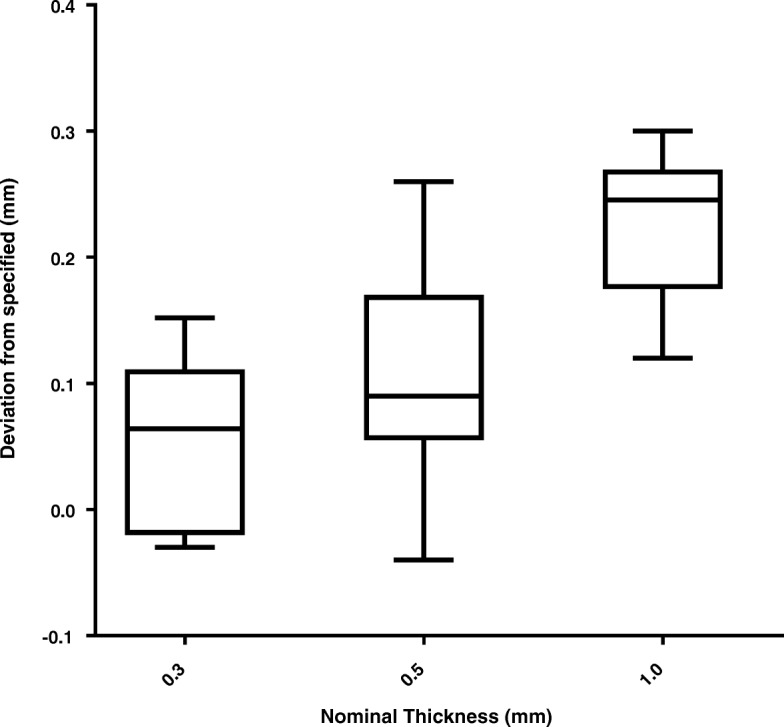
Box plot of SLM production accuracy for different nominal thicknesses of disk

Deviations from the nominal disk thickness are summarised in Fig. 5. There was no significant correlation between printing method and thickness accuracy. Production accuracy ranged from − 40 to 300 μm with an average deviation of 20% from the nominal thicknesses set within the central section of the disk.

### Attenuation measurements

**Fig. 6:**
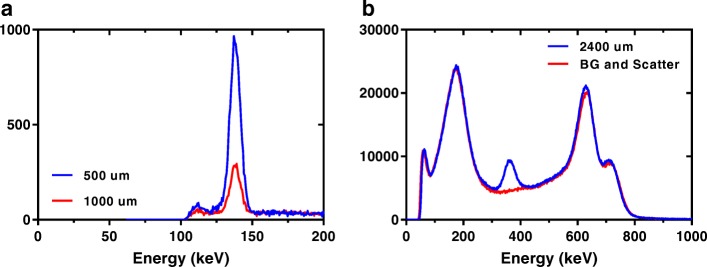
Obtained energy spectra of **a** Tc-99m and **b** I-131 source through different thicknesses of Tungsten

Example energy spectra for the collimated Tc-99 m and I-131 sources are shown in Fig. 6 through different thicknesses of tungsten. A significant amount of scatter and penetration is observed through the lead shield from the high-energy photons (> 600 keV) of the I-131 spectra.

**Fig. 7:**
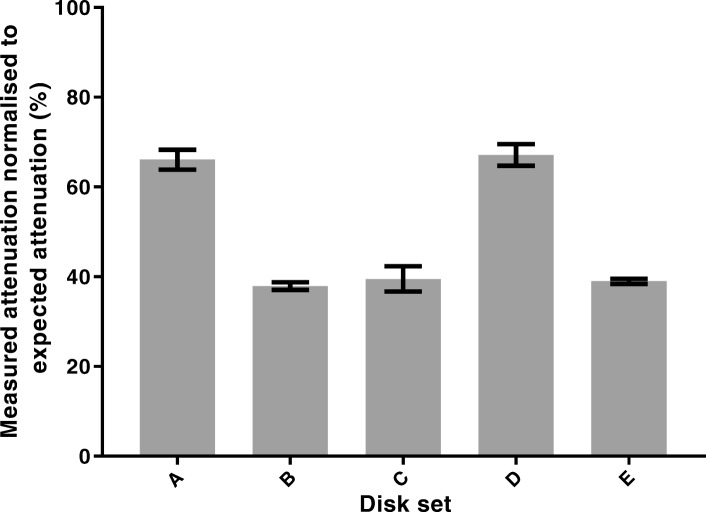
Beam attenuation measured through 0.5-mm-thick disks produced with different scan strategies

Of the five disk sets manufactured, sets B and C showed visible signs of cracking at thicknesses below 1.0 mm and an insufficient number of disks were available for an adequate measurement of μ. However, with the disks available, a measure of attenuation through 0.5 mm of each tungsten disk could be determined. Figure 7 shows the total beam attenuation of 140 keV photons through a 0.5 mm tungsten disk of each sample set. Results are expressed as a percentage of the expected attenuation based on NIST data given in Table 3. Where multiple disks from each set were available, results were repeated and the standard deviation expressed as error bars on the chart.

Of the five sets of disks, A and D demonstrated approximately 70% of the expected attenuation. Disks from sets B, C and E performed less well with only 40% of the expected attenuation.

**Fig. 8:**
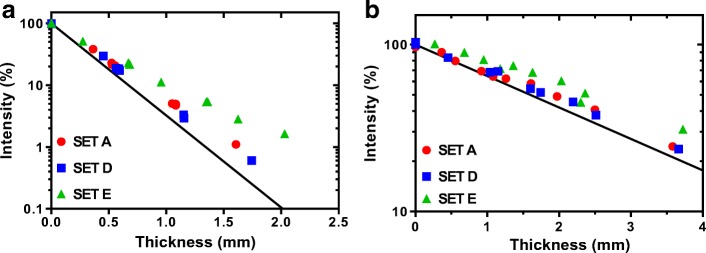
Beam intensity through different thicknesses of SLM tungsten printed with different scan strategies and different beam intensities **a** 140 keV, **b** 364 keV compared to the theoretical ideal (solid line)

**Fig. 9:**
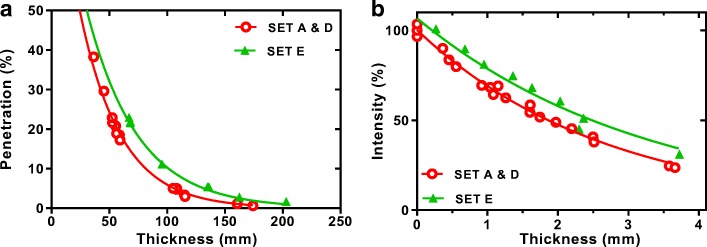
Beam intensity through different thicknesses of SLM tungsten printed with different scan strategies and different beam intensities **a** 140 keV, **b** 364 keV data fitted using least squares regression

Plots of normalised count rate (intensity) as a function of total tungsten disk thickness for each disk set measured at 140 keV and 364 keV are shown on log-linear plots in Fig. 8. The solid line on each figure shows the theoretical ideal. It can be seen that all three sets of disks have lower attenuation properties than the theoretical prediction. Non-linear regression fits to the data are shown on a linear scale in Fig. 9a, b. An extra sum-of-squares *F* test was used to determine if there was any significant difference between fit coefficients for each disk set. Measurements with Tc-99m and I-131 showed no significant difference between disk sets A and D characterised by an *F* statistic (DFn, DFd) = 0.066 (1,15), *P* value = 0.0001, but a statistically significant difference from E, with an *F* statistic (DFn, DFd) = 20.95 (1,24), *P* value = 0.80. A summary of the measured attenuation coefficient for each disk set compared to the theoretical ideal values are summarised in Table 6.

**Table 6:** Measured and attenuation properties of disks

Material	Measured μ (cm^−1^)	% deviation from theoretical (including Coherent scatter)	% deviation from theoretical (excluding Coherent scatter)
Samples A and D (140 keV)	28.1 ± 0.3	22–23%	17–19%
Sample E (140 keV)	22.7 ± 0.4	36–38%	33–35%
Samples A and D (364 keV)	3.7 ± 0.1	12–17%	5–10%
Sample E (364 keV)	3.1 ± 0.2	24–33%	18–28%

### Collimator measurements

**Fig. 10:**
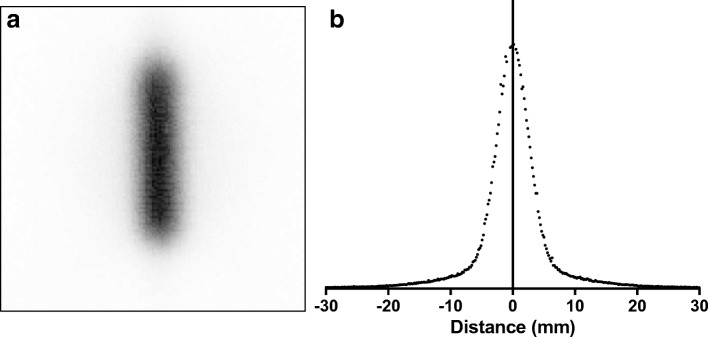
Line source image (**a**) and profile (**b**)

The acquired line source image using the tungsten collimator is shown in Fig. 10a, and a summed line profile through the 20 cm central region of the source is given in Fig. 10b. The “tails” at either side of the peak are a result of scatter and septal penetration within the collimator. Ten single pixel width line profiles at intervals down the length of the source were also analysed. FWHM measurements of each profile were measured and an extra sum-of-squares *F* test demonstrated no significant difference (*P* value = 0.14), indicating a uniform resolution across the length of the source (i.e. no image distortion or significant variation in septal thicknesses or hole diameter).

**Fig. 11:**
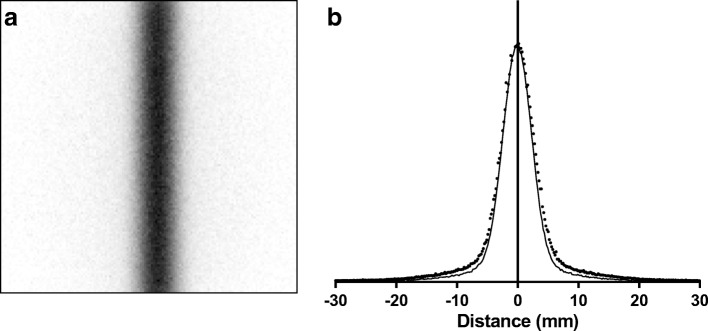
Monte Carlo simulated line source image (**a**) and line profile compared to measured (**b**). Simulated assuming 100% density and nominal septal thickness

Monte Carlo simulated images of the line source, using the nominal specified geometry for pure tungsten at 100% density, are shown in Fig. 11a. Line profiles through the Monte Carlo image overlaid on the physical measurements are shown in Fig. 11b. It can be seen that the amount of scatter and septal penetration is slightly underestimated in the simulation.

**Fig. 12:**
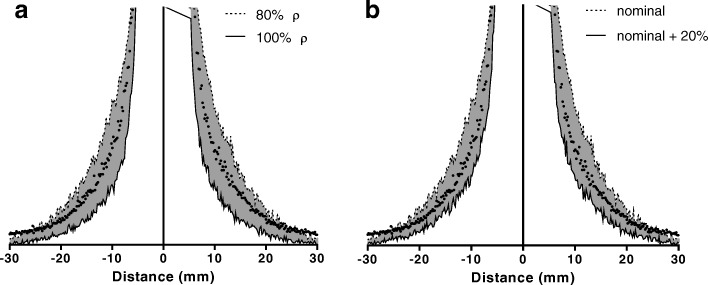
Monte Carlo simulated line profiles compared to measured, for different material densities (**a**) and septa thicknesses (**b**)

A line profile simulated for tungsten at 80% and 100% density is compared to the measured data in Fig. 12a. The scale of the plot has been reduced to show the difference in the scatter tails of the data. Similar profiles are shown in Fig. 12b, for 80% density and septal thicknesses of 0.2 mm (nominal) and 0.24 mm (nominal + 20%).

**Fig. 13:**
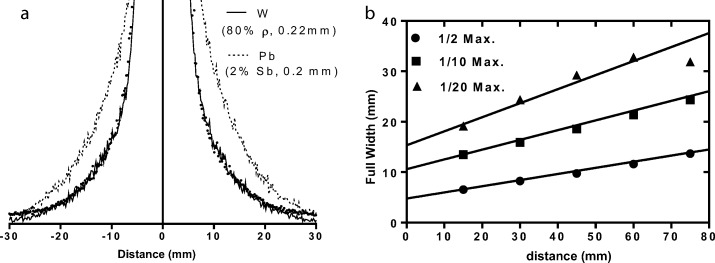
Monte Carlo simulated line profiles of tungsten (80% ρ, 0.22 mm septa) and lead (25Sb, 0.2 mm septa) collimators (**a**) and comparison of measured (points) and simulated (solid line) (tungsten 80% ρ, 0.22 mm septa) resolution at different distances (**b**)

The optimal match to measured data was found to be 80% density and + 10% additional septal thickness. Comparison of this simulation with measured data and a similar lead collimator are shown in Fig. 13. Measurements of the full width half, tenth and twentieth maximums at different distances for the physical and simulated collimator are shown in Fig. 13b. A slight deviation is observed between the measured and simulated profiles at the tails, which is beyond the physical edge of the phantom and thought to be due to additional scatter through the lead shield surrounding the collimator. This scatter is negligible compared to peak counts in the profiles and in general, a good match for all full width measurements is observed.

The numerical significance of the reduced density is summarised in Table 7, using results generated from the Monte Carlo simulation. The matched collimator is highlighted in grey. The percentages of photons within the line source image that have scattered or penetrated are shown against those that have passed through geometrically. The effect of the additional septal thickness is demonstrated in the geometric efficiency which is 6% lower for the matched collimator.

**Table 7:** Simulated components of scatter within energy window for different collimator materials and designs

Material	Tungsten			Lead (2% Sb)
Density (g/cm)	100	80	80	100
Septal thickness (mm)	0.20	0.20	0.22	0.20
Geometrical collimation	86.7%	80.6%	82.2%	79.2%
Penetration through septa	6.7%	9.1%	8.9%	10.2%
Scatter within collimator	6.6%	10.3%	8.9%	10.6%
Relative geometric efficiency	100%	100%	94%	100%

## Discussion

SLM is a complex process with a number of potential variables that could affect the performance of SLM manufactured tungsten collimators. Production accuracy, as a measure against nominal disk thickness, is an indication of the potential variation that may be experienced in septal thickness when manufacturing collimators. In this work, a deviation of − 40 to 300 μm was measured against the nominal thickness. These values are comparable to that reported by Deprez et al. who observed variations of − 260 to 650 μm. Duprez et al. took measurements from a diverging collimator; the diverging nature of the septa from the printing plane could explain the slightly larger variation observed compared to our study. The impact of such deviation is dependent on the nominal thickness, and in this work the mean deviation was 20% (range − 8% to 52%). The surface of the material is not perfectly smooth, and the physical measurements of thickness are based on the extremities of the surface which may partially account for observed variations. Measurements of larger pieces (such as the physical collimator size) see no significant difference between specified and produced object sizes.

Direct measurements of attenuation of Tc-99 m and I-131 through lead disks were made. Measurements were used to determine the linear attenuation coefficient, results of which agreed to within 4% of the theoretical value for Tc99m and 10% of the theoretical value for I-131. The higher variation observed with I-131 is likely due to the higher amount of scatter present in the I-131 spectra (Fig. 6) and although a scatter correction was applied, isolating penetrating photons from scattered photons is difficult and the correction may not be 100%. However, the results with Tc-99m were considered sufficient to validate the measurement technique.

During manufacture of the SLM tungsten, two sample sets showed visible signs of cracking. This can be explained by the scanning strategy employed for these disks. Table 1 shows pictorially the scanning paths which for these disks are parallel to the short dimension of the disk. Furthermore, this path is repeated on subsequent layers. The distance between laser paths is known as the hatch spacing and it follows that a weakening can exist where these paths overlap, particularly for very short path lengths.

**Fig. 14:**
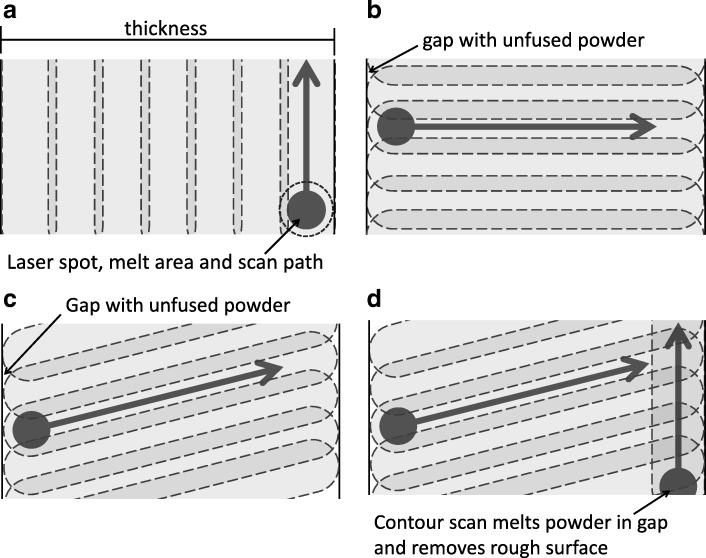
Schematic illustration of scan strategies, showing laser spot, melt area, path direction and overlap. **a** Scan path is perpendicular to object face. **b** Scan path is parallel to object face. **c** Scan path is angled from object face. **d** Scan path is angled with the addition of contour scan around periphery of object

Measurements of attenuation through 0.5 mm samples of SLM tungsten are shown in Fig. 7. It can be seen that three of the samples (B, C and E) demonstrate reduced attenuation properties compared to samples A and D. This result was further examined by measuring the attenuation coefficients for sets A, D and E, where it was shown (Table 5) that all samples demonstrated attenuation properties lower than the theoretical ideal and that a further significantly reduced attenuation coefficient of sample E was observed. Results of these attenuation measurements indicate that either the samples are not comprised of pure tungsten, or the density of the SLM material is significantly lower than the raw tungsten density. An apparent density of the tungsten can be determined by linearly scaling the measured attenuation with the theoretical values. For the difference in attenuation observed, samples A and D performed similarly to that of 80% density and sample E performed similarly to that of 65% density. Direct measurements of SLM tungsten density have previously been carried out with reported ranges from 80 to 96% [2–4]. The reduction in density in the literature is generally attributed to microscopic air cavities in the material between the fused metal particles. The standard approach for density determination in these examples is to measure the sample mass using a precision balance and sample volume using the Archimedes suspension method. The difficulty with applying this approach is that for a precise measurement, a bulk piece of material is required. It does not follow that a similar density will be achieved when printing smaller parts or that the density of the material is uniform across a printed object. Therefore, a significant difference between previous measurements and those published here is that we were able to measure attenuation through the SLM material produced in the same scan geometry that a collimator would be printed. Deprez et al. have also made direct measurements of SLM tungsten attenuation. In this case, they measured attenuation through tungsten plates, cut from an SLM-printed block and therefore the photon beam and print direction would not necessarily be the same orientation as in a collimator design. Results from our investigation demonstrate that this could have a significant impact on the material density. Additionally, the surface texture of the SLM tungsten is removed during the electric wire discharge machining used to cut the plates from the block. This is significant because surface “roughness” will also account for the apparent reduction in density and for the differences observed between disk samples. Figure 14 illustrates the laser spot scan path for different scanning strategies. In each case, the laser melts an area of tungsten slightly larger than the spot size, and the hatch spacing is sufficient that there is an overlap on each scan path. In Fig. 14a for a scan path perpendicular to the measured thickness (equivalent to scan strategy A), it can be seen that the laser melt area covers the whole thickness of the object. Conversely in Fig. 14b, c (equivalent to scan strategy B and E), a gap is observed at the edge of the object, which is not covered by the laser path. In Fig. 14d, a contour scan is applied and the gaps at the surface are removed (equivalent to scan strategy D).

**Fig. 15:**
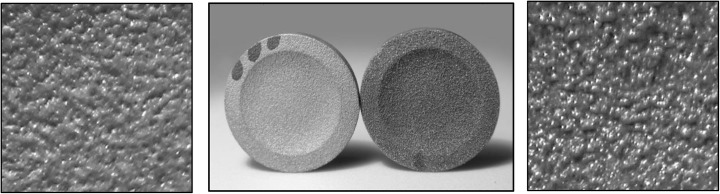
Photographs of disks produced with (left) and without (right) contour scan

This hypothesis explains the improved attenuation properties of sets A and D over that of the other scan strategies. Furthermore, the textural difference in scan patterns can be observed visually. Figure 15 is a photograph of the disks produced using scan strategies D and E where the absence of the contour scan is clearly apparent.

**Fig. 16:**
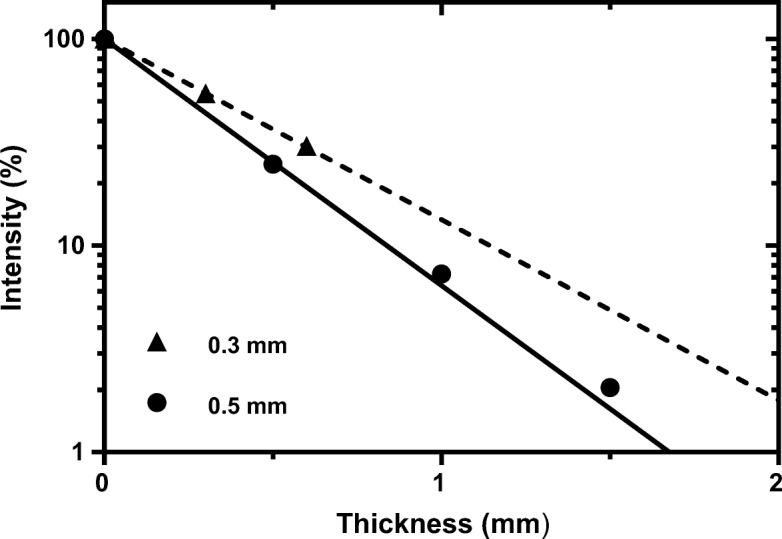
Beam intensity through SLM tungsten disks printed at different nominal thicknesses

The implications of “surface roughness” will vary depending on the object thickness, scan direction and spot size, and will become negligible for bulk objects. However, the importance at small thicknesses should not be overlooked. This is demonstrated in Fig. 16 where an apparent difference is observed between 0.5 and 0.3 mm disks (although statistically not significant due to the small number of 0.3 mm disks).

The effect of reduced attenuation properties of the SLM tungsten were highlighted in the collimator acquisitions. The “tails” at either side of the line source and the non-Gaussian shape of the line profile indicate scatter and septal penetration within the collimator. Iterative Monte Carlo simulations of differing collimator properties indicated that the SLM collimator was a similar match to 80% density tungsten and 110% nominal septal thickness. These results are comparable to the deviations observed in the measurements of μ and thickness determined in the disk experiments. Results from the simulations suggest that 33% more scatter and penetration occurs in the collimator as a result of the reduced attenuation properties, compared to “pure” 100% density tungsten. Equally, the SLM collimator exhibits 17% less scatter and penetration that the nominal lead collimator. However, in each case, the total scatter to geometric photons is low and the total difference is less than 4.5% of the total photons within the energy window.

Due to the apparent reduced density of SLM tungsten, attenuation performance of the material is less than the theoretical density (assuming 100% density). However, with careful selection of scanning strategies and a knowledge of material performance, design optimisation can be achieved. Additional optimisation may also be achieved by further investigation of additional scan parameters such as laser powder, scanning speed and hatch distance. Microstructure analysis would also be useful to help understand the differences from each scan strategy and also to analyse some possible defects like cracking, porosity and a lack of fusion that will impact on the material performance.

Measured attenuation properties indicate that shielding could be reduced by 10% when replacing lead with SLM tungsten. Performance of SLM tungsten has been shown to be superior to lead alternatives and the unique opportunity for bespoke designs. Current manufacture technology is limited by the size of the powder bed chamber, which for an EOS M280 results in a maximum foot print of 250 mm by 300 mm and maximum collimator thickness of 200 mm. This current limitation restricts the use of SLM to small FOV collimators, such as for portable and small animal scanners. However, as technology advances and larger printing chambers become available, clinical-sized collimators could become a possibility; alternatively, collimators tessellation could be investigated to increase the FOV.

The reproducibility of collimator production has not been assessed in this study. For 0.5-mm-thick disks, produced using scan strategy D, the coefficient of variation of disk thickness was 2.7% and it is therefore possible that a similar variation in collimator septa could also be present (although no image distortion or significant variation in image resolution was observed). It is also possible that collimator production may vary between print batches. To assess batch reproducibility, the performance characteristics of identical collimators manufactured at different times would have to be analysed. In addition, further variation may be observed across different SLM machines and it is therefore advisable that adequate acceptance tests are performed on all novel collimator designs produced using SLM. In addition, a thorough quality control procedure should be performed if this technology is used for mass production. Imaging application of SLM collimators will likely be most suited to high-energy isotopes, such as I-131 where thicker septa would be less affected by surface roughness.

## Conclusions

The attenuation properties of SLM tungsten have been characterised. SLM tungsten was shown to have superior attenuation properties compared to lead and a valid alternative for collimator design. The surface texture of the tungsten can have a significant impact on apparent material density and careful selection of the scanning strategy is required when using SLM to ensure optimum design performance.

## Abbreviations

NIST: National Institute of Standards and Technology
SLM: Selective laser melting
